# Study on the Effect of Basalt Fiber on the Mechanical Properties of Recycled Micro Powder Mortar

**DOI:** 10.3390/ma19040764

**Published:** 2026-02-15

**Authors:** Weidong Cheng, Xinzhong Wang, Xianliang Tan, Yuexing Wu, Yuwen Sun, Biao Zhou, Yi Xiang, Linshu Li

**Affiliations:** College of Civil Engineering, Hunan City University, Yiyang 413000, China; 2403y01-18@stu.hncu.edu.cn (W.C.); tanxianliang@hncu.edu.cn (X.T.); wuyuexing@hncu.edu.cn (Y.W.); 2403y01-08@stu.hncu.edu.cn (Y.S.); 18373777035@163.com (B.Z.); xiangyi@hncu.edu.cn (Y.X.); lilinshu@hncu.edu.cn (L.L.)

**Keywords:** recycled micro-powder, basalt fiber, flowability, mechanical properties, early strength prediction model

## Abstract

To improve the workability and mechanical properties of Recycled Micro-Powder (RP) mortar, basalt fiber (BF) was used for modification in this study. Experimental groups with different BF contents (0%, 0.1%, 0.15%, and 0.2%) were designed to investigate the effects of BF on the flowability, flexural strength, and compressive strength of RP mortar. The microscopic reinforcement mechanism was further revealed through Scanning Electron Microscopy (SEM). Additionally, an early strength prediction model for the mortar considering the synergistic effect of BF and RP was established. The results show that the incorporation of BF significantly enhanced the mechanical properties of RP mortar. At the 28-day curing age, when the RP replacement rate was 10% and BF content was 0.15%, the flexural strength increased by 9.7%, and the compressive strength increased by 17.3%. At an RP replacement rate of 30%, the compressive strength still increased by over 30%, demonstrating a good “performance compensation” effect. However, the inclusion of BF also led to a decrease in flowability, with a maximum reduction of 25.5%. SEM analysis revealed that BF improved the matrix densification and interface bonding performance through crack bridging and physical anchoring. The established early strength prediction model achieved a high goodness of fit (R^2^ > 0.92), indicating high accuracy and engineering applicability.

## 1. Introduction

The environmental issues caused by construction waste are becoming increasingly severe worldwide, and finding reasonable methods to process and utilize construction waste has become an important challenge faced by China and many other countries [1]. Currently, the recycling of construction waste is primarily focused on the development and application of recycled aggregates, while Recycled Micro-Powder (RP), with a particle size smaller than 75 μm, is still generally disposed of through stacking or landfilling, leading to resource waste and environmental pollution [2,3,4]. Therefore, using recycled micro-powder as a partial replacement for cement in cementitious materials has become a key method for achieving high-value utilization of construction waste.

However, the cementitious activity and particle characteristics of recycled micro-powder are significantly inadequate [5]. Its high specific surface area and rough surface texture increase the water demand, reduce the flowability of the slurry, and weaken the interfacial bonding performance, severely affecting the mechanical properties of mortars and concrete [6,7,8,9]. Previous studies have shown that when the substitution rate of recycled micro-powder exceeds 10%, both the flowability and compressive strength of the mortar significantly decline [10,11,12,13,14,15]. Therefore, improving the flowability and strength of recycled micro-powder mortar through composite modification technologies has become a key issue in current research on recycled building materials. It should be emphasized that, in recycled micro-powder composite systems, workability and mechanical performance often exhibit a mutually restrictive trade-off relationship, and the underlying contradiction between them still lacks systematic investigation.

Fiber reinforcement technology is an important method for improving the performance of cement-based materials [16,17,18,19,20,21]. Among them, basalt fiber (BF), compared with steel fibers and some polymer fibers, is characterized by a fine diameter, a moderate elastic modulus, and relatively high surface roughness. Under relatively low fiber dosages, basalt fibers can provide effective crack-bridging and restraining effects, showing the potential to enhance mechanical performance while relatively alleviating adverse effects on workability [22,23,24,25,26,27]. Studies by Wang Liankun et al. [28], Wu Jinan et al. [29], and Wang Y.G. et al. [30] have shown that the incorporation of basalt fiber can significantly improve the flexural strength, compressive strength, and toughness of ordinary mortar and concrete. However, most existing research focuses on the application of BF in ordinary mortar or recycled aggregate concrete, with limited systematic studies on its rheological behavior, mechanical properties, and microscopic reinforcement mechanisms in recycled micro-powder mortar systems. Meanwhile, most existing studies have adopted admixtures or mix proportion adjustment methods to mitigate the workability reduction caused by fiber incorporation. Under conditions without introducing additional adjustment measures, fundamental research on the degree of workability deterioration induced by fiber incorporation in recycled micro-powder mortar and its relationship with mechanical performance enhancement remains insufficient.

In addition, most existing studies have employed recycled micro-powder derived from actual demolished construction waste. Such materials usually exhibit complex compositions, high impurity contents, and significant source variability, which are unfavorable for quantitative analysis of material behavior. Based on this consideration, laboratory-generated waste concrete was used in this study to prepare recycled micro-powder in order to reduce the interference caused by raw material source variability and impurities, thereby enabling a clearer elucidation of the synergistic interaction mechanisms between recycled micro-powder and basalt fibers. It should be emphasized that the present study is positioned as a fundamental investigation focusing on basic performance and mechanisms, and the obtained conclusions are intended to provide a reference for subsequent engineering application studies employing recycled micro-powder derived from real demolition waste.

Therefore, this study takes basalt fiber-reinforced recycled micro-powder mortar as the research object. Without introducing chemical admixtures or adjusting the basic mix proportions, the effects of different BF dosages on the flowability, flexural strength, and compressive strength of recycled micro-powder mortar are investigated. The focus is placed on revealing the trade-off relationship between workability degradation and mechanical performance enhancement, rather than achieving engineering-level compensation of workability through admixtures or mix proportion modification. Furthermore, scanning electron microscopy (SEM) is employed to elucidate the microstructural reinforcement mechanisms of basalt fibers. On this basis, an early-age strength prediction model considering the synergistic effects of BF and RP is established to provide theoretical support and data reference for the performance optimization and engineering application of recycled micro-powder mortar, particularly in non-structural cementitious components, masonry mortars and overlay layers, as well as repair and strengthening applications [31,32].

## 2. Materials and Methods

### 2.1. Materials and Preparation Process

The experimental materials include sand, mixing water, cement, recycled micro-powder from waste concrete, and basalt fiber.

Sand: ISO standard sand produced by a standard sand factory.

Mixing Water: Tap water.

Cement: Ordinary Portland cement P·O 42.5, produced by Southern Cement Co., Ltd. (Changsha, China).

**Figure 1:**
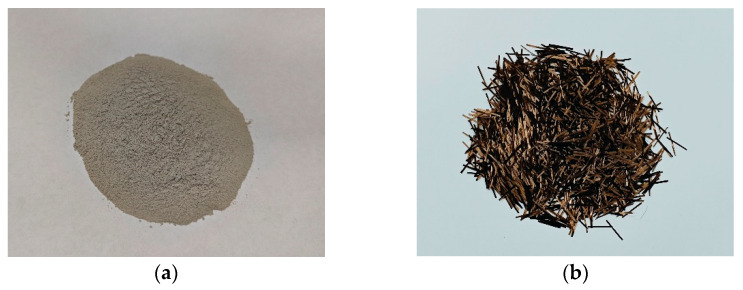
Experimental materials: (**a**) recycled micro-powder; (**b**) basalt fiber.

Recycled Micro-Powder (RP): The recycled micro-powder (Figure 1a) used in this experiment was sourced from laboratory waste concrete specimens. Waste concrete with a strength grade of C30 was crushed and sieved to obtain micro-powder with a particle size smaller than 75 μm. Laboratory-generated waste concrete was adopted to minimize the variability in raw material composition and the interference of impurities, thereby enabling a clearer investigation of the fundamental interactions between recycled micro-powder and basalt fibers.

Basalt Fiber (BF): Short-cut basalt fiber (Figure 1b) produced by Zhejiang Shijin Basalt Fiber Co., Ltd. (Jinhua, China), with a size range of 17 μm to 12 mm, The physical properties are shown in Table 1. This fiber diameter was selected because fine-diameter basalt fibers are more suitable for mortar-scale matrices, allowing effective crack-bridging and stress transfer at relatively low dosages, while reducing the risk of severe fiber entanglement. The chemical composition and surface characteristics of basalt fiber were further characterized by SEM–EDS analysis, as summarized in Table 2. 

**Figure 2:**
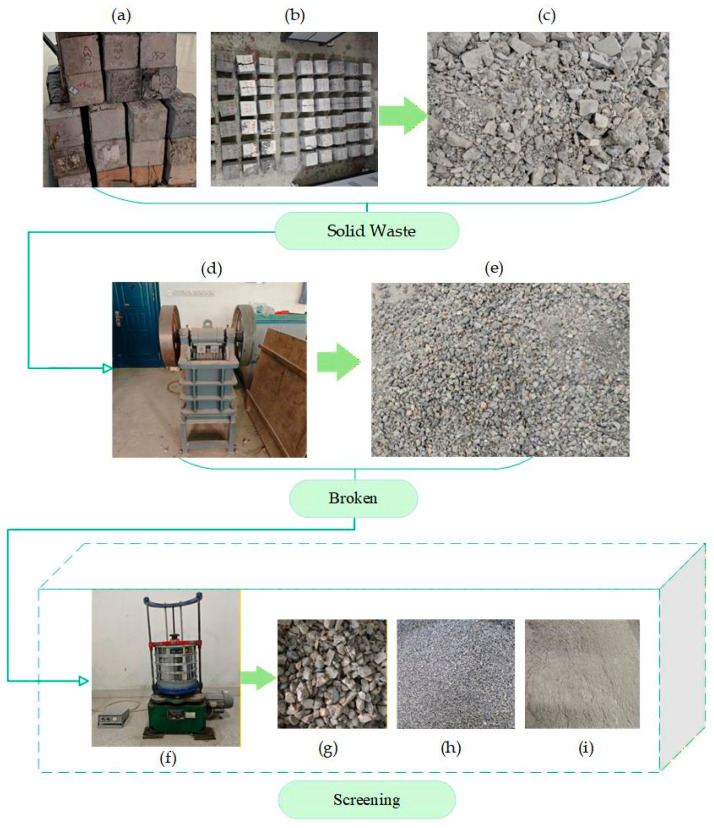
Preparation process of recycled micro-powder derived from laboratory-generated waste concrete: (**a**) cured waste concrete blocks; (**b**) laboratory-collected waste concrete specimens prepared for crushing; (**c**) concrete fragments after primary crushing; (**d**) laboratory jaw crusher used for crushing; (**e**) crushed concrete after secondary crushing; (**f**) mechanical sieving apparatus; (**g**) coarse aggregate fraction; (**h**) fine aggregate fraction; (**i**) recycled micro-powder with particle size smaller than 75 μm.

The recycled micro-powder from waste concrete is a gray-brown powdered material, and the chemical composition of the cementitious material is shown in Table 3. To prepare recycled micro-powder that meets the experimental requirements, two aspects of quality control were applied. The first aspect is the control of particle size range. The particle size of the micro-powder was controlled according to the specifications in “Recycled Fine Aggregates for Concrete and Mortar” (GB/T 25176—2010) [33], ensuring the particle size is less than 75 μm. The second aspect is the control of fineness, which was measured and controlled according to “Test Methods for Cement Fineness” (GB/T 1345—2005) [34] (the preparation process of recycled micro-powder is shown in Figure 2).

### 2.2. Mix Proportion Design

A total of 16 groups of specimens were designed in this study, with curing ages of 3, 7, and 28 days. The mix proportions of RP cement mortar are presented in Table 4, including three different RP replacement ratios. The basalt fiber content was defined based on the volume fraction relative to the total volume of the mortar mixture. According to Test Method for Strength of Cement Mortar (ISO method) (GB/T 17671–2021) [35], the reference strength grade of cement mortar was M30. All materials were mixed following standardized procedures. During the mixing process, basalt fibers were gradually introduced into the fresh mixture to reduce the possibility of fiber agglomeration. All mixtures were prepared using a fixed mixing sequence and mixing duration to ensure consistency and comparability.

After casting, all mortar specimens were demolded after 24 h. Subsequently, the specimens were cured in a standard curing room at a temperature of 20 ± 2 °C and a relative humidity higher than 95% for 3, 7, and 28 days. After the designated curing periods, the specimens were removed for mechanical testing and microstructural analysis.

No chemical admixtures, such as plasticizers or superplasticizers, were incorporated in this study. All mixtures were prepared using identical mix proportions and mixing procedures, in order to directly evaluate the intrinsic influence of recycled micro-powder and basalt fiber incorporation on workability and mechanical performance.

### 2.3. Mortar Performance Testing

The flowability of the mortar was tested according to the Chinese National Standard “Test method for fluidity of cement mortar” (GB/T 2419-2005) [36], using a standard flow table apparatus (model NLD-3). The specimens were prismatic, with dimensions of 40 mm × 40 mm × 160 mm. Mechanical performance testing was conducted following the “Test Method for Strength of Cement Mortar (ISO method)” (GB/T 17671-2021) [35]. During the molding process, a stainless-steel three-part mold was used in combination with a vibration table (frequency 50 Hz, amplitude 0.5 mm) to compact the mortar, ensuring consistency and reliability in the specimen structure. After molding, the specimens were cured in a standard curing room at a temperature of (20 ± 1) °C and a relative humidity not lower than 95% for 24 h before demolding. They were then further cured under the same conditions for 3, 7, and 28 days.

**Figure 3:**
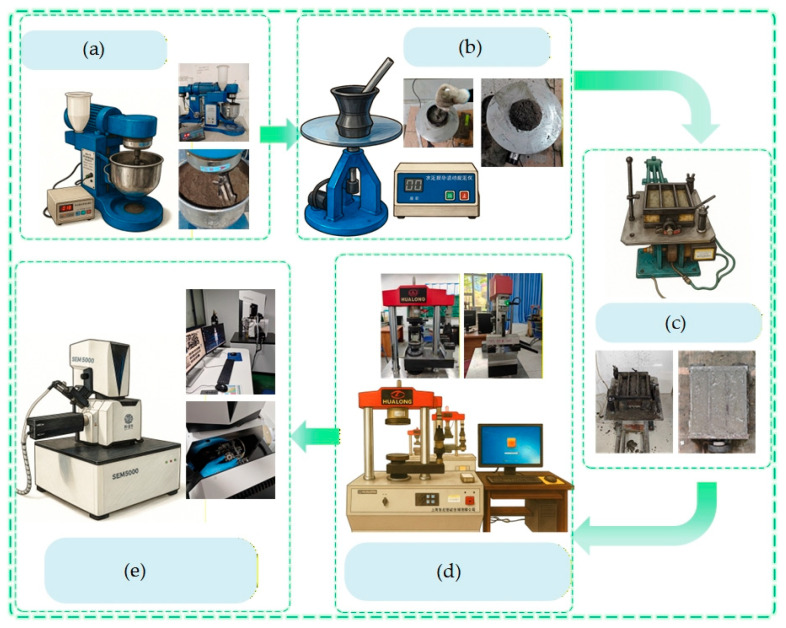
Experimental Setup Diagram: (**a**) Mortar mixer; (**b**) Flow meter; (**c**) Vibration table; (**d**) Pressure and bending resistant all-in-one machine; (**e**) SEM5000 Scanning Electron Microscope.

The flexural strength was tested using a three-point bending method, carried out with a microcomputer-controlled electro-hydraulic servo universal testing machine (model WAW-1000) (Shanghai Hualong, Shanghai, China). Compressive strength was tested using a specialized compressive fixture (contact area: 40 mm × 40 mm) at a loading rate of 2.4 kN/s, ensuring a smooth loading process and data comparability. To further analyze the microstructure and morphological characteristics of the hydration products, representative samples were selected, the hydration was terminated using ethanol, and the samples were vacuum-dried before undergoing Scanning Electron Microscopy (SEM) observation. The experimental setup is shown in Figure 3, ensuring that the testing procedures comply with standard requirements and offer good repeatability.

## 3. Results and Discussion

For each test condition, at least three parallel specimens were prepared, and the reported results represent the average values. The relative variation among parallel tests was within an acceptable range, indicating good repeatability of the experimental results.

### 3.1. Flowability Analysis

**Figure 4:**
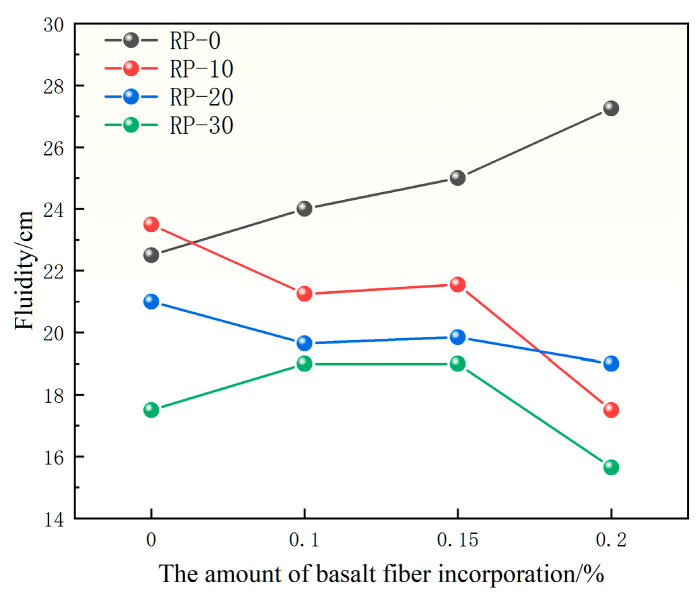
Effect of BF content on the flowability of RP mortar.

Figure 4 shows the effect of basalt fiber (BF) content on the flowability of mortar with different recycled micro-powder (RP) substitution rates. Overall, the incorporation of basalt fiber has a noticeable influence on the flowability of the mortar, and a decreasing trend can be observed as the fiber content increases [37,38].

Under a fixed RP substitution rate, the flowability of recycled micro-powder mortar exhibits a consistent decreasing trend as the BF content increases from 0% to 0.2%. When the RP substitution rate is 10%, the flowability decreases from 23.5 cm to 17.5 cm, corresponding to an average reduction of approximately 25.5%. At an RP substitution rate of 20%, the flowability decreases from 21.0 cm to 19.0 cm, with a reduction of about 9.5%. At an RP substitution rate of 30%, a more pronounced decrease is observed, with the flowability decreasing from 17.5 cm to 15.65 cm. Although experimental variability exists, the observed reduction in flowability shows a consistent trend across different RP substitution levels.

The reduction in flowability can be attributed to the increased internal resistance of the fresh mixture associated with fiber incorporation, as the presence of basalt fibers increases the overall surface area and hinders the free movement of the mortar matrix.

In conclusion, the incorporation of basalt fiber is associated with a reduction in the flowability of recycled micro-powder mortar, and this effect becomes increasingly evident with higher fiber contents and RP substitution rates. Nevertheless, under the investigated mix proportions, the measured flowability values remain within a range generally considered acceptable for practical mortar applications

### 3.2. Mechanical Performance Analysis

#### 3.2.1. Flexural Strength Analysis

**Figure 5:**
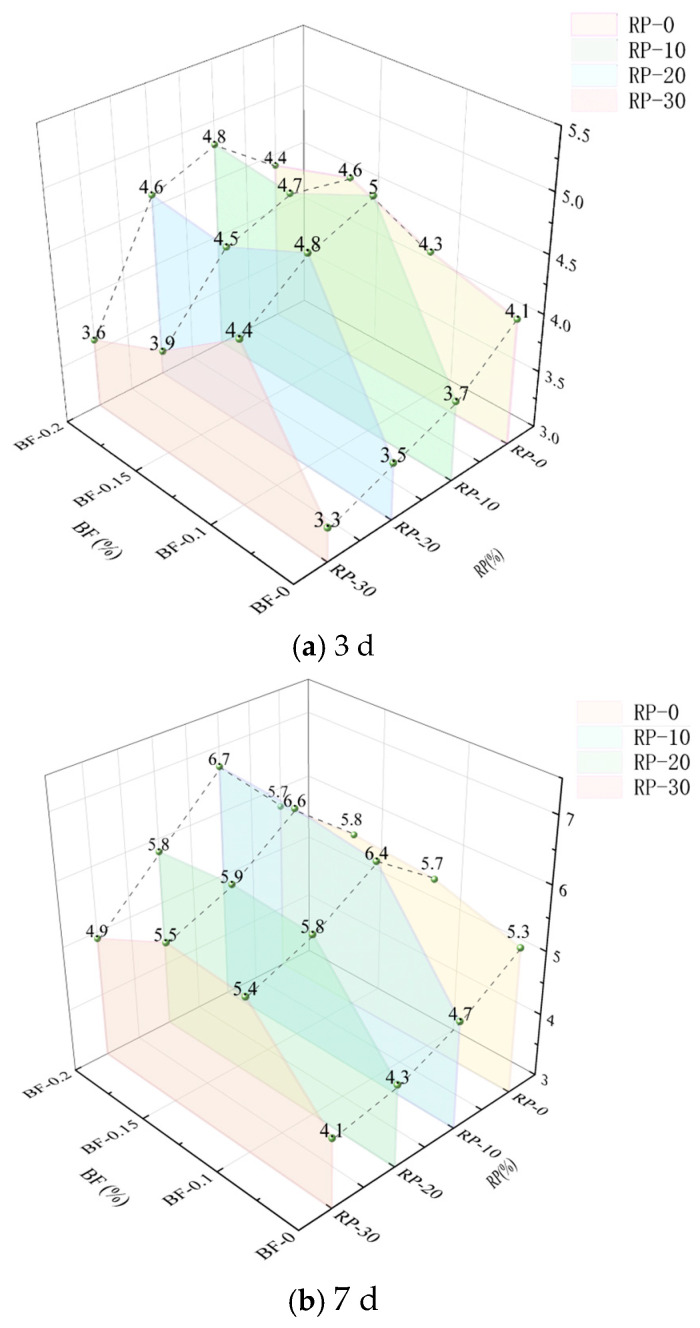

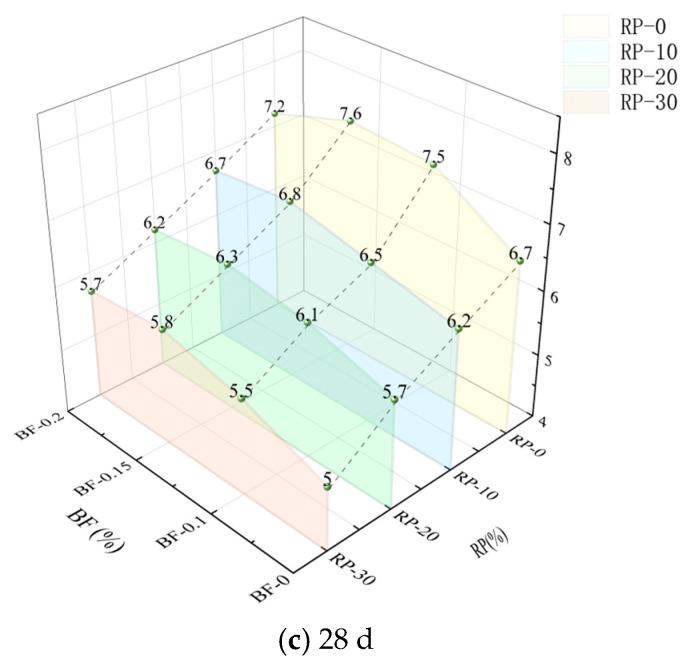
Effect of BF content on the flexural strength of RP mortar at different curing ages.

To systematically evaluate the effect of Basalt Fiber (BF) on the flexural performance of Recycled Powder (RP) cement mortar, flexural strength tests were conducted on mortar samples with different combinations of BF and RP contents at 3, 7, and 28 days of curing. The results are shown in Figure 5a–c. The results indicate that the incorporation of basalt fiber is associated with an improvement in the flexural strength of the mortar at all curing ages, and this enhancement shows an increasing trend with curing time, with relatively favorable performance observed at 28 days.

At 3 days of curing (Figure 5a), the overall strength of the mortar was relatively low. However, after the introduction of BF, the flexural strength of all specimens exhibited an increasing trend, with a comparatively more evident enhancement observed at BF = 0.15%. When the RP substitution rate was 10%, the flexural strength increased from 3.7 MPa (without fiber) to 4.6 MPa, corresponding to an average increase of 24.3%. This suggests that the contribution of BF to flexural performance begins to manifest even at early curing ages, although the mortar matrix is still in the early stages of development.

At 7 days of curing (Figure 5b), the strength of all groups further increased. With RP = 0 and BF = 0, the flexural strength was 5.3 MPa. As curing progressed, the mortar matrix became denser, and the reinforcing effect associated with fiber incorporation became more apparent. Compared to the 3-day results, the enhancement in flexural strength associated with BF showed a more pronounced trend. With RP = 10% and BF = 0.15%, the flexural strength reached 6.7 MPa, the highest among all specimens, representing an average increase of 42.4% compared to the BF = 0 group. Furthermore, at an RP substitution rate of 30%, the incorporation of BF exhibited a noticeable compensating effect, partially alleviating the reduction in flexural strength associated with higher RP contents.

At 28 days of curing (Figure 5c), the strength of the mortar tended to stabilize, and the reinforcing effect associated with BF incorporation became more evident. With RP = 10% and BF = 0, the flexural strength was 6.2 MPa, and when the BF content was 0.15%, it increased to 6.8 MPa, corresponding to an average improvement of 9.7%. With RP = 30%, the flexural strength increased from 5.0 MPa (without BF) to 5.8 MPa (with BF = 0.15%), representing an average increase of approximately 16%. Overall, the enhancement in flexural strength associated with BF incorporation shows clear age dependence and sensitivity to fiber content, with the strengthening effect becoming more evident as curing progresses, and relatively favorable performance observed at a BF content of 0.15% under the investigated conditions.

Furthermore, the flexural strength exhibits a trend of first increasing and then slightly decreasing with increasing BF content. At all RP levels, the flexural strength increases as BF content increases from 0% to 0.15%. However, when the BF content reaches 0.2%, a slight reduction in flexural strength is observed in some groups, which may be associated with fiber agglomeration or changes in the interfacial transition zone quality [26,39]. These results suggest that controlling the BF content within an appropriate range is beneficial for improving flexural performance, while excessive fiber incorporation may reduce the effectiveness of reinforcement.

#### 3.2.2. Compressive Strength Analysis

**Figure 6:**
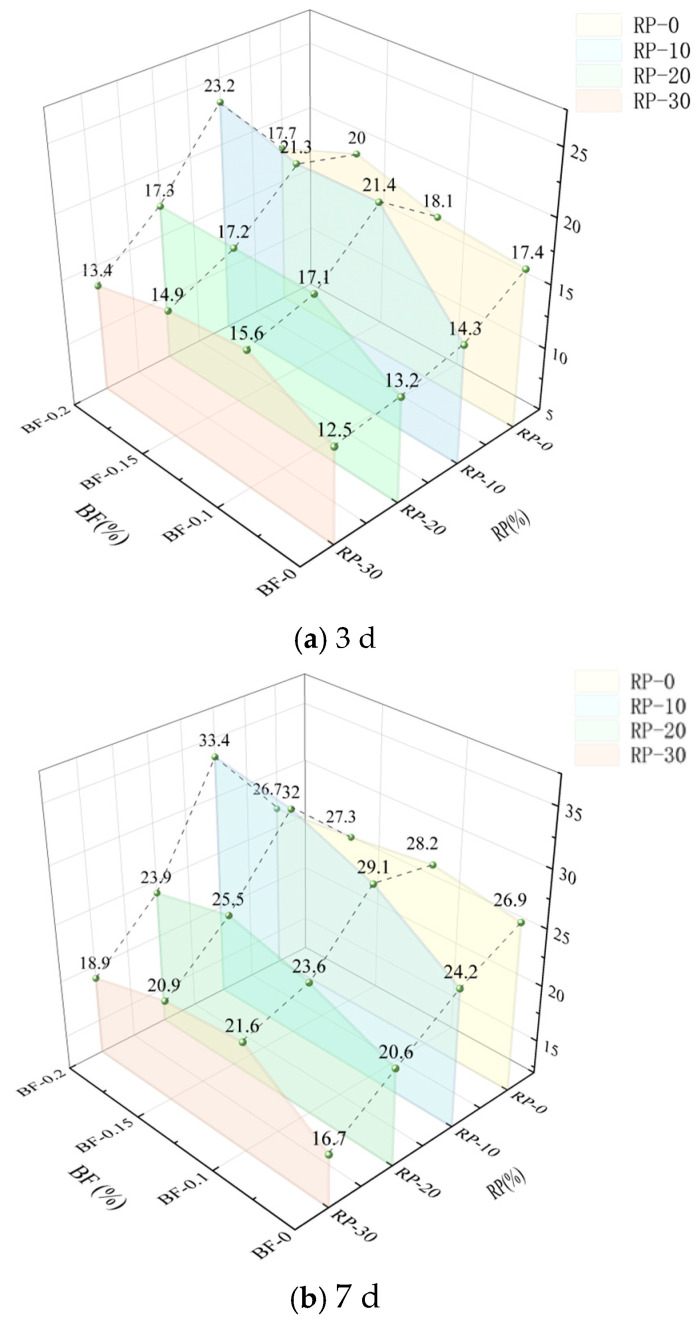

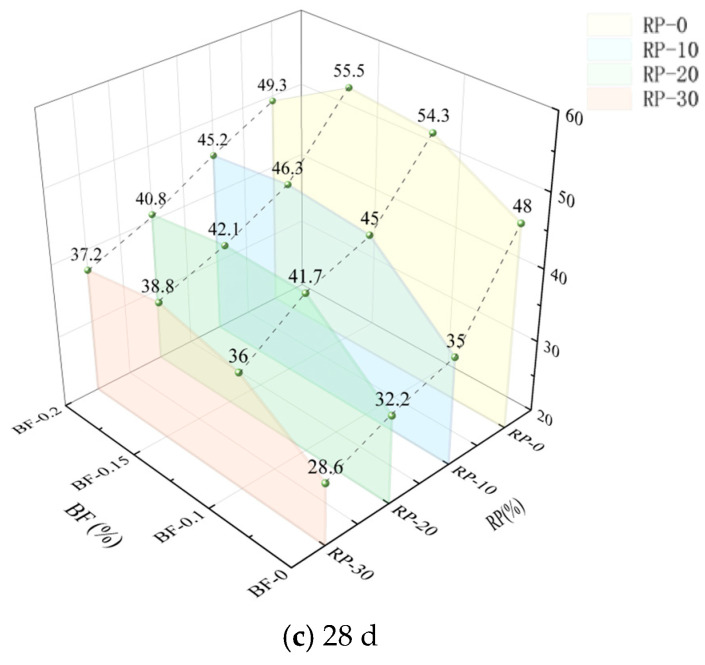
Effect of BF content on the compressive strength of RP mortar at different curing ages.

Figure 6 shows the variation in compressive strength of Basalt Fiber (BF) at different Recycled Powder (RP) mortar conditions at 3 d, 7 d, and 28 d curing ages. Overall, the compressive strength of the mortar exhibits a clear increasing trend with curing age. At the same curing age, the compressive strength is influenced by both RP and BF contents, exhibiting a nonlinear trend characterized by an initial increase followed by a slight decrease at higher fiber contents.

At 3 days of curing (Figure 6a), the recycled powder mortar is in the early stage of structural development, and the compressive strength is generally low. However, after the introduction of BF, the compressive strength of all specimens shows an increasing trend, with a comparatively more evident enhancement observed at BF = 0.15%. At RP = 10%, the compressive strength reached 21.3 MPa, representing the highest average value at this curing age, corresponding to an average increase of 48.9% compared to the fiber-free group. This suggests that BF incorporation contributes to early-age strength development, possibly by restricting the propagation of micro-cracks and improving the integrity of the forming structure.

At 7 days of curing (Figure 6b), the compressive strength of the mortar further increased, reflecting the continued formation of hydration products and progressive densification of the matrix. For RP = 10% and BF = 0, the compressive strength was 24.2 MPa, while at BF = 0.15%, it increased to 32.0 MPa, corresponding to an average improvement of 32.2%. At a higher RP substitution rate of 30%, the compressive strength of the BF = 0 group was 16.7 MPa, whereas with BF incorporation it increased to 20.9 MPa, representing an average increase of 25.1%. These results indicate that BF incorporation can partially compensate for the reduction in compressive strength associated with higher RP contents.

At 28 days of curing (Figure 6c), the compressive strength of all specimens tended to stabilize, corresponding to a more fully developed hardened structure. The strengthening effect associated with BF incorporation becomes more evident at this stage. For RP = 0, the compressive strength increased from 48.0 MPa for the BF = 0 group to 55.5 MPa for the BF = 0.15% group, corresponding to an average increase of 15.6%. In the RP = 10% group, the compressive strength increased from 46.3 MPa to 54.3 MPa, representing an average improvement of 17.3%. Even at a high RP substitution rate of 30%, the compressive strength increased from 28.6 MPa (BF = 0) to 37.2 MPa (BF = 0.15%), corresponding to an average increase of approximately 30.1%. Overall, BF incorporation is associated with an enhanced load-bearing capacity of the mortar at later curing ages, particularly under higher RP substitution conditions.

Across all curing ages, the influence of BF content on compressive strength exhibits an effective content range. Under the investigated experimental conditions, a BF content of 0.15% shows relatively favorable enhancement effects across most RP substitution levels. When the BF content was further increased to 0.2%, a slight reduction in compressive strength was observed in some specimens, which may be associated with fiber agglomeration and localized discontinuities in the matrix [25,26]. These observations suggest that excessive fiber incorporation may reduce reinforcement efficiency, emphasizing the importance of controlling BF content within an appropriate range.

### 3.3. Microstructure Mechanism Analysis of Recycled Powder Cement Mortar

**Figure 7:**
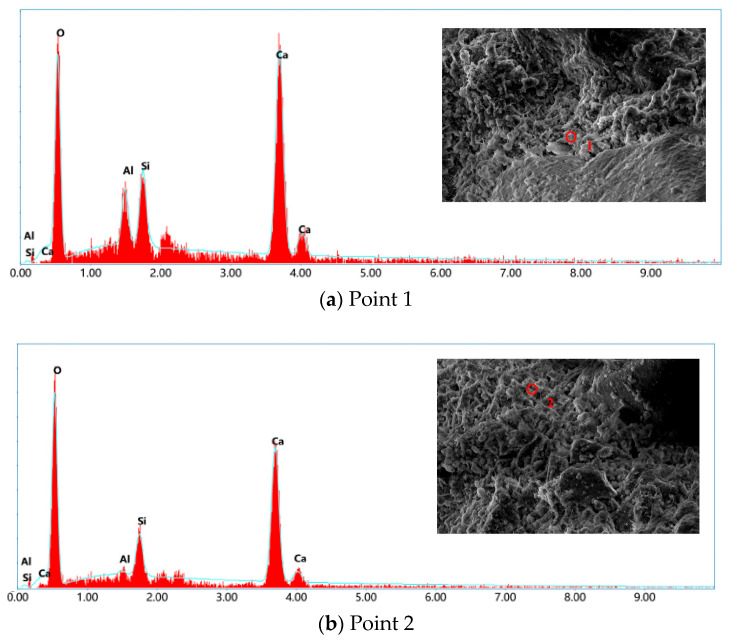
EDS spectrum.

**Figure 8:**
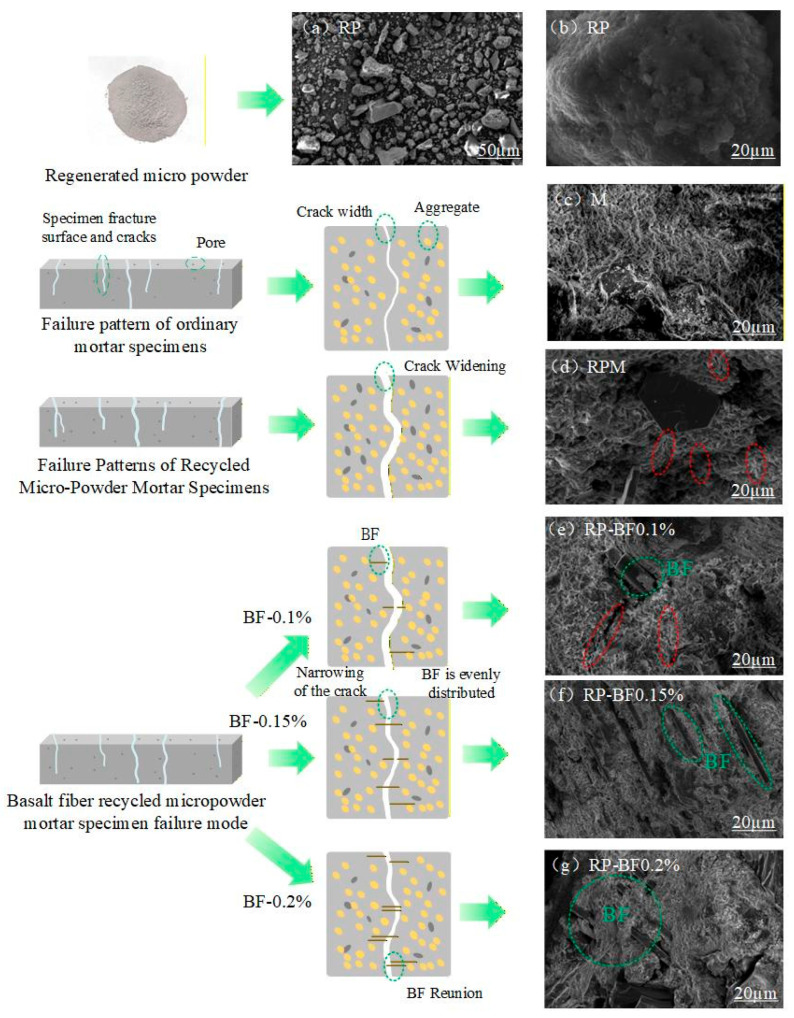
Microstructural Mechanism of Basalt Fiber-Recycled Powder Mortar and SEM Scanning Electron Microscopy Analysis: In the SEM images, the green circles indicate the fiber–matrix bridging zones, while the red circles represent the microcrack initiation regions.

To further reveal the microstructural characteristics associated with the incorporation of Basalt Fiber (BF) in modified Recycled Powder (RP) mortar, representative samples were selected and observed using Scanning Electron Microscopy (SEM). The results are shown in Figure 7 and Figure 8.

Figure 8a,b show that the RP particles are irregular in shape, with rough surfaces and a relatively high presence of pores and micro-cracks. Such morphological features are commonly associated with limited apparent pozzolanic contribution under the investigated conditions, which may contribute to insufficient matrix densification and relatively weak interfacial bonding, thereby being associated with a reduction in mechanical performance. To complement the morphological observations, SEM–EDS analysis was conducted to provide qualitative information on the elemental composition of RP, as shown in Figure 7 and summarized in Table 5. The EDS results indicate that RP is mainly composed of O, Si, Ca, and Al, with minor amounts of Mg, Fe, Na, and K. This elemental composition is consistent with the typical characteristics of recycled powders derived from waste concrete, which generally consist of hydrated cementitious materials and residual aggregate phases. It should be noted that the SEM–EDS analysis is intended to support basic compositional characterization rather than to quantitatively evaluate the chemical reactivity of RP [40].

Figure 8c presents the fracture surface morphology of ordinary cement mortar (M), where the matrix appears relatively dense and the hydration products are more uniformly distributed. After the incorporation of recycled micro-powder (Figure 8d), the fracture surface becomes comparatively rougher, with a higher presence of unhydrated particles and pore structures, and a more tortuous crack path. These observations suggest that recycled micro-powder alters the crack propagation path within the matrix. In addition, some residual hydration products associated with the recycled micro-powder may act as nucleation sites or fillers, contributing to partial pore refinement. However, limited interfacial bonding under the investigated conditions may still lead to local debonding between micro-powder particles and the cement paste.

With the incorporation of basalt fiber (Figure 8e–g), noticeable changes in the microstructural features of the mortar are observed. In Figure 8e, when the BF content is 0.1%, the fibers appear relatively well distributed within the matrix, and a close contact between the fibers and cement paste can be observed at the interface. This distribution suggests a potential crack-bridging effect, as indicated by locally reduced crack widths. At this stage, the contribution of BF is likely associated with micro-crack bridging and stress redistribution, whereby the fibers may bear part of the tensile stress and delay crack propagation.

When the BF content increases to 0.15% (Figure 8f), the fiber distribution appears more uniform, and the fiber–matrix interface becomes more compact. Hydration products, such as C–S–H gels, can be observed adhering to the fiber surface, which suggests an enhanced interfacial interaction. The presence of fibers may promote local densification in the interfacial transition zone, and the observed cracks are finer and more branched, indicating an improved resistance to crack propagation under these conditions.

However, when the BF content further increases to 0.2% (Figure 8g), localized fiber agglomeration is observed. Such fiber accumulation may introduce micro-pores and voids, which can disrupt the continuity of the matrix and create local stress concentration zones. These microstructural features may partially offset the reinforcing effect of the fibers, leading to a reduction in overall compactness and mechanical performance.

In conclusion, SEM observations suggest that a BF content of 0.15% is associated with a relatively uniform internal structure, a more compact fiber–matrix interface, and refined crack morphology under the investigated conditions, which may contribute to improved crack resistance and matrix densification. It should be noted that the densification behavior discussed here is inferred from microstructural observations, and further quantitative characterization (e.g., porosity or density measurements) will be considered in future work.

### 3.4. Early Strength Prediction Analysis

The establishment of an early strength prediction model is beneficial for engineers in practical construction processes. Accurate prediction of early concrete strength helps engineers optimize the concrete mix design. Existing early compressive strength prediction models for recycled concrete have primarily focused on the use of recycled coarse aggregates [41,42], as shown in Equation $f_{c,t}=f_{c,28}\cdot \frac{\mathrm{lg}t}{\mathrm{lg}28}$. Based on experimental data for basalt fiber contents of 0%, 0.1%, 0.15%, and 0.2%, and RP substitution rates of 0%, 10%, 20%, and 30%, the flexural strengths at 3 d, 7 d, and 28 d were extracted. On this basis, an early flexural and compressive strength prediction model for mortar incorporating both recycled micro-powder and basalt fiber was developed, as shown in Equations (1) and (2).

(1) $$\frac{f_{\mathrm{flex},t}}{f_{\mathrm{flex},28}}=a\cdot \frac{\mathrm{lg}t}{\mathrm{lg}28}+b$$

(2) $$\frac{f_{c,t}}{f_{c,28}}=a\cdot \frac{\mathrm{lg}t}{\mathrm{lg}28}+b$$

where

*f*_flex,t_: Flexural strength at curing age t (t = 3, 7) in MPa;
*f*_flex,28_: Flexural strength at 28-day curing age in MPa;
*f*_c,t_: Compressive strength at curing age t (t = 3, 7) in MPa;
*f*_c,28_: Compressive strength at 28-day curing age in MPa;
a, b: Regression coefficients.

The a and b values for the 16 replacement groups were fitted. The coefficient of determination (R^2^) is primarily used to evaluate the explanatory power of the regression model for the dependent variable. Its value ranges from 0 to 1, with R^2^ values closer to 1 indicating a better fit of the regression model. Generally, R^2^ > 0.75 is considered to reflect a high degree of model fit and strong explanatory capability, while R^2^ < 0.50 suggests that linear regression may not be appropriate for the data.

The fitted a and b values were used as dependent variables, and the RP replacement rate (α) and basalt fiber content (β) were taken as independent variables. Flexural and compressive strength data under different BF contents (BF = 0, 0.1, 0.15, 0.2) and RP replacement rates (0%, 10%, 20%, 30%) were normalized and substituted into Equations (3) and (4) for fitting. In these equations, ei, fi, and hi are fitting parameters. The resulting regression parameters are presented in Table 6.

**Figure 9:**
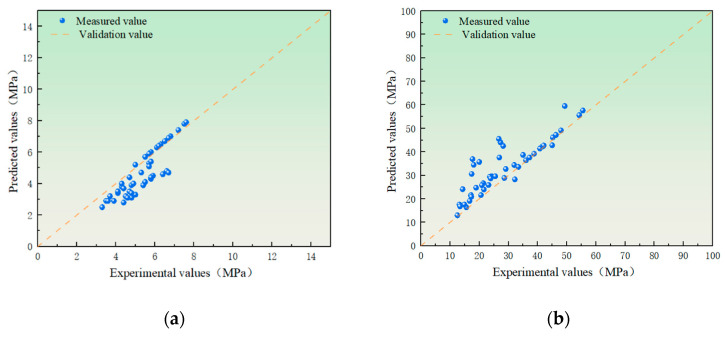
Comparison between experimental and predicted values for strength fitting: (**a**) flexural strength fitting results; (**b**) compressive strength fitting results.

As shown in Figure 9, the model exhibits excellent fitting performance, with all R^2^ values exceeding 0.92, indicating strong applicability and predictive capability of the developed models.

a = e_1_ × α + f_1_ × β + h_1_ (3)

b = e_2_ × α + f_2_ × β + h_2_ (4)

By substituting the parameters from Table 6 into Equations (3) and (4), the early flexural strength and compressive strength prediction models for the combined use of recycled micro-powder and basalt fiber can be obtained, as shown in Equations (5) and (6).

(5) $$\frac{f_{\mathrm{flex},t}}{f_{\mathrm{flex},28}}=\left(0.0082\alpha +1.245\beta +0.780\right)\cdot \frac{\mathrm{lg}t}{\mathrm{lg}28}+(-0.0155\alpha -0.720\beta +0.253)$$

(6) $$\frac{f_{c,t}}{f_{c,28}}=\left(0.841\alpha +0.025\beta +0.576\right)\cdot \frac{\mathrm{lg}t}{\mathrm{lg}28}+(-0.880\alpha -0.025\beta +0.447)$$

## 4. Conclusions

(1) This study investigated the combined effects of recycled micro-powder (RP) substitution and basalt fiber (BF) incorporation on the fresh and mechanical properties of cement mortar under controlled mix proportions.

(2) The incorporation of basalt fiber resulted in a reduction in mortar flowability, and this effect became more pronounced with increasing BF content and RP substitution rate. Within the investigated range, the maximum reduction in flowability reached 25.5%. Nevertheless, under all tested mix proportions, the measured flowability values remained within a range generally considered acceptable for practical mortar applications.

(3) The flexural and compressive strengths of recycled micro-powder mortar increased with curing age at 3, 7, and 28 days. Basalt fiber incorporation enhanced mechanical performance across different RP substitution levels. At an RP substitution rate of 10% and a BF content of 0.15%, the 28-day compressive strength increased from 46.3 MPa to 54.3 MPa (17.3%), while the flexural strength increased from 6.7 MPa to 7.6 MPa (13.4%). Within the investigated experimental range, a BF content of 0.15% provided the most favorable strength performance under most RP substitution conditions, whereas further increases in BF content resulted in no additional strength gain or slight reductions in some cases.

(4) From an engineering perspective, recycled micro-powder mortars modified with basalt fiber are suitable for non-structural cementitious applications, including masonry mortars, surface rendering and overlay layers, floor screeds, and repair or strengthening works, where adequate strength and crack resistance are required.

## Figures and Tables

**Table 1:** Physical and mechanical properties of basalt fiber.

Material	Density (g/cm^3^)	Breaking Strength (MPa)	Breaking Elongation (%)	Elastic Modulus (GPa)	Tensile Strength (MPa)
BF	≥2.6	≥2000	2.0~3.0	85	4200

**Table 2:** Basalt fiber EDS elemental composition (wt%).

Element	O	Si	Al	Ca	Mg	Fe	Na	K
Point 1	47.2	23	8	7.4	4.4	6.8	1.9	0.7
Point 2	46.5	23.4	8.3	7.7	4.1	7	1.8	0.6
Point 3	47	22.8	8.1	7.5	4.3	7.2	1.9	0.6
Average	46.9	23.07	8.13	7.53	4.27	7	1.87	0.63
Std. dev.	0.36	0.31	0.15	0.15	0.15	0.2	0.06	0.06

**Table 3:** Chemical composition of cementitious materials (%).

Material	CaO	SiO_2_	Al_2_O_3_	Fe_2_O_3_	MgO	SO_3_	Others
Cement	59.85	19.80	5.53	3.39	1.12	—	10.31
Recycled Micro-Powder	30.76	38.41	13.07	10.56	4.00	1.21	1.99

**Table 4:** Recycled micronized mortar mix ratio.

Experimental Group	Fiber (% by Volume)	Recycled Micro-Powder Substitution Rate (%)	Cement (g)	Water/Cement Ratio	Standard Sand (g)	Water (g)
BF-0-RP-0	0	0	450	0.5	1350	225
BF-0-RP-10	0	10	405	0.5	1350	225
BF-0-RP-20	0	20	360	0.5	1350	225
BF-0-RP-30	0	30	315	0.5	1350	225
BF-0.1-RP-0	0.10	0	450	0.5	1350	225
BF-0.1-RP-10	0.10	10	405	0.5	1350	225
BF-0.1-RP-20	0.10	20	360	0.5	1350	225
BF-0.1-RP-30	0.10	30	315	0.5	1350	225
BF-0.15-RP-0	0.15	0	450	0.5	1350	225
BF-0.15-RP-10	0.15	10	405	0.5	1350	225
BF-0.15-RP-20	0.15	20	360	0.5	1350	225
BF-0.15-RP-30	0.15	30	315	0.5	1350	225
BF-0.2-RP-0	0.20	0	450	0.5	1350	225
BF-0.2-RP-10	0.20	10	405	0.5	1350	225
BF-0.2-RP-20	0.20	20	360	0.5	1350	225
BF-0.2-RP-30	0.20	30	315	0.5	1350	225

**Table 5:** Surface elemental composition of RP (wt%).

Element	O	Si	Al	Ca	Mg	Fe	Na	K	Ti
Point 1	45.9	26.6	7.4	11.7	1.9	2.6	2.1	1.1	0.7
Point 2	47.2	25.1	6.3	13.1	2.4	2	1.5	1.7	0.7

**Table 6:** Fitting parameters and R^2^.

Value of Each Parameter		e_i_	f_i_	h_i_	R^2^
Folding fit parameters	a	0.0082	1.245	0.780	0.932
	b	−0.0155	−0.720	0.253	0.961
Compression fitting parameters	a	0.841	0.025	0.576	0.924
	b	−0.880	−0.025	0.447	0.966

## Data Availability

The original contributions presented in this study are included in the article. Further inquiries can be directed to the corresponding author.
